# Adherence to the World Cancer Research Fund/American Institute for Cancer Research cancer prevention recommendations and risk of in situ breast cancer in the European Prospective Investigation into Cancer and Nutrition (EPIC) cohort

**DOI:** 10.1186/s12916-019-1444-0

**Published:** 2019-12-02

**Authors:** Nena Karavasiloglou, Anika Hüsing, Giovanna Masala, Carla H. van Gils, Renée Turzanski Fortner, Jenny Chang-Claude, Inge Huybrechts, Elisabete Weiderpass, Marc Gunter, Patrick Arveux, Agnès Fournier, Marina Kvaskoff, Anne Tjønneland, Cecilie Kyrø, Christina C. Dahm, Helene Tilma Vistisen, Marije F. Bakker, Maria-Jose Sánchez, María Dolores Chirlaque López, Carmen Santiuste, Eva Ardanaz, Virginia Menéndez, Antonio Agudo, Antonia Trichopoulou, Anna Karakatsani, Carlo La Vecchia, Eleni Peppa, Domenico Palli, Claudia Agnoli, Salvatore Panico, Rosario Tumino, Carlotta Sacerdote, Salma Tunå Butt, Signe Borgquist, Guri Skeie, Matthias Schulze, Timothy Key, Kay-Tee Khaw, Kostantinos K. Tsilidis, Merete Ellingjord-Dale, Elio Riboli, Rudolf Kaaks, Laure Dossus, Sabine Rohrmann, Tilman Kühn

**Affiliations:** 1 Division of Chronic Disease Epidemiology, Institute for Epidemiology, Biostatistics and Prevention, University of Zurich, Zurich, Switzerland; 2 Cancer Registry Zurich and Zug, University Hospital Zurich, Zurich, Switzerland; 3 Division of Cancer Epidemiology, German Cancer Research Center (DKFZ), Heidelberg, Germany; 4 Cancer Risk Factors and Life-Style Epidemiology Unit, Institute for Cancer Research, Prevention and Clinical Network - ISPRO, Florence, Italy; 5 Julius Center for Health Sciences and Primary Care, University Medical Center Utrecht, Utrecht University, Utrecht, The Netherlands; 6 International Agency for Research on Cancer, Lyon, France; 7 Department of Community Medicine, University of Tromsø, The Arctic University of Norway, Tromsø, Norway; 8 CESP, Fac. de médecine - Univ. Paris-Sud, Fac. de médecine - UVSQ, INSERM, Université Paris-Saclay, 94805 Villejuif, France; 9 Gustave Roussy, F-94805 Villejuif, France; 10 Breast and Gynaecologic Cancer Registry of Côte d’Or, Georges-François Leclerc Cancer Centre, UNICANCER, Dijon, France; 11 Department of Public Health, Faculty of Health and Medical Sciences, University of Copenhagen, Copenhagen, Denmark; 12 Danish Cancer Society Research Center, Copenhagen, Denmark; 13 Department of Public Health, Aarhus University, Aarhus, Denmark; 14 Andalusian School of Public Health (EASP), Granada, Spain; 15 Instituto de Investigación Biosanitaria de Granada (ibs. GRANADA), Granada, Spain; 16 CIBER of Epidemiology and Public Health (CIBERESP), Madrid, Spain; 17 Universidad de Granada, Granada, Spain; 18 Department of Epidemiology, Murcia Regional Health Council, IMIB-Arrixaca, Murcia, Spain; 19 Department of Health and Social Sciences, Murcia University, Murcia, Spain; 20 Navarra Public Health Institute, Pamplona, Spain; 21 IdiSNA, Navarra Institute for Health Research, Pamplona, Spain; 22 Public Health Directorate, Asturias, Spain; 23 Unit of Nutrition and Cancer, Catalan Institute of Oncology - ICO, Nutrition and Cancer Group, Bellvitge Biomedical Research Institute - IDIBELL, L’Hospitalet de Llobregat, Barcelona, Spain; 24 Hellenic Health Foundation, Athens, Greece; 25 2nd Pulmonary Medicine Department, School of Medicine, “ATTIKON” University Hospital, National and Kapodistrian University of Athens, Haidari, Greece; 26 Department of Clinical Sciences and Community Health, Università degli Studi di Milano, Milan, Italy; 27 Epidemiology and Prevention Unit, Fondazione IRCCS Istituto Nazionale dei Tumori, Milan, Italy; 28 Department of Clinical Medicine and Surgery, Federico II University, Naples, Italy; 29 Cancer Registry and Histopathology Department, Azienda Sanitaria Provinciale (ASP), Ragusa, Italy; 30 Unit of Cancer Epidemiology, Città della Salute e della Scienza University-Hospital and Center for Cancer Prevention (CPO), Turin, Italy; 31 Department of Clinical Sciences, Lund University, Malmö, Sweden; 32 Department of Surgery, Skåne University Hospital, Malmö, Sweden; 33 Department of Oncology, Aarhus University Hospital, Aarhus University, Aarhus, Denmark; 34 Division of Oncology and Pathology, Clinical Sciences, Lund University, Lund, Sweden; 35 Nutritional Epidemiology Group, School of Food Science and Nutrition, University of Leeds, Leeds, UK; 36 Department of Molecular Epidemiology, German Institute of Human Nutrition Potsdam-Rehbrücke (DIfE), Nuthetal, Germany; 37 Nuffield Department of Population Health, University of Oxford, Oxford, UK; 38 Department of Public Health and Primary Care, University of Cambridge, Cambridge, UK; 39 Department of Epidemiology and Biostatistics, School of Public Health, Imperial College London, London, UK; 40 Department of Hygiene and Epidemiology, University of Ioannina School of Medicine, Ioannina, Greece

**Keywords:** In situ breast cancer, Cohort, Lifestyle, Prevention, Lifestyle Score

## Abstract

**Background:**

Even though in situ breast cancer (BCIS) accounts for a large proportion of the breast cancers diagnosed, few studies have investigated potential risk factors for BCIS. Their results suggest that some established risk factors for invasive breast cancer have a similar impact on BCIS risk, but large population-based studies on lifestyle factors and BCIS risk are lacking. Thus, we investigated the association between lifestyle and BCIS risk within the European Prospective Investigation into Cancer and Nutrition cohort.

**Methods:**

Lifestyle was operationalized by a score reflecting the adherence to the World Cancer Research Fund/American Institute for Cancer Research (WCRF/AICR) cancer prevention recommendations. The recommendations utilized in these analyses were the ones pertinent to healthy body weight, physical activity, consumption of plant-based foods, energy-dense foods, red and processed meat, and sugary drinks and alcohol, as well as the recommendation on breastfeeding. Cox proportional hazards regression was used to assess the association between lifestyle score and BCIS risk. The results were presented as hazard ratios (HR) and corresponding 95% confidence intervals (CI).

**Results:**

After an overall median follow-up time of 14.9 years, 1277 BCIS cases were diagnosed. Greater adherence to the WCRF/AICR cancer prevention recommendations was not associated with BCIS risk (HR = 0.98, 95% CI 0.93–1.03; per one unit of increase; multivariable model). An inverse association between the lifestyle score and BCIS risk was observed in study centers, where participants were recruited mainly via mammographic screening and attended additional screening throughout follow-up (HR = 0.85, 95% CI 0.73–0.99), but not in the remaining ones (HR = 0.99, 95% CI 0.94–1.05).

**Conclusions:**

While we did not observe an overall association between lifestyle and BCIS risk, our results indicate that lifestyle is associated with BCIS risk among women recruited via screening programs and with regular screening participation. This suggests that a true inverse association between lifestyle habits and BCIS risk in the overall cohort may have been masked by a lack of information on screening attendance. The potential inverse association between lifestyle and BCIS risk in our analyses is consistent with the inverse associations between lifestyle scores and breast cancer risk reported from previous studies.

## Background

In situ breast cancer (BCIS) is an intraepithelial lesion with malignant potential. Generally, it is considered a non-obligatory precursor or a potential risk factor for invasive breast cancer. Women with BCIS rarely report symptoms, and the majority of in situ tumors are detected through organized or opportunistic mammographic screening attendance [1].

Even though BCIS accounts for up to 20% of the breast cancer cases diagnosed [2], its prognosis and possible etiological similarities with invasive breast cancer remain unclear. In epidemiological studies on breast cancer risk, in situ tumors are often excluded [3], censored [4], or included together with invasive tumors as a composite endpoint [5]. Only few studies have investigated potential risk factors for BCIS suggesting that some established risk factors for invasive breast cancer have a similar impact on BCIS risk [6–9]. Regarding lifestyle factors, alcohol consumption and smoking were found to be associated with a non-statistically significant trend for increased risk of BCIS in postmenopausal women [6], whereas higher body mass index (BMI) was associated with a statistically significant decreased risk for BCIS in premenopausal women [7]. Physical activity studies showed mixed findings, with some reporting no association with BCIS risk [10, 11], while others reported an inverse association [12]. However, comprehensive analyses on pre-diagnostic diet and lifestyle in relation to BCIS from large-scale cohort studies are sparse.

Considering the abovementioned studies suggesting an inverse association of a more health-conscious lifestyle with a decreased risk of BCIS, and the lack of large epidemiological studies, we investigated the association between lifestyle, operationalized by a score reflecting the adherence to cancer prevention recommendations, and BCIS risk within the European Prospective Investigation into Cancer and Nutrition (EPIC) cohort.

## Methods

### Study population and data collection

EPIC is a multicenter prospective cohort study designed to investigate the associations between diet, lifestyle, genetic and environmental factors, and the incidence of cancers and other chronic diseases. Over 520,000 participants, aged 25–70 years, were recruited between 1992 and 2000 in 10 European countries (Denmark, France, Germany, Greece, Italy, the Netherlands, Norway, Spain, Sweden, and the UK). The French cohort included members of a health insurance plan for school and university employees. Participants at the Spanish and Italian centers (except Florence) included blood donors, members of several health insurance programs, employees of several enterprises, civil servants, and people from the general population. In Utrecht and Florence, women mainly participating in mammographic screening programs were recruited for the study. In Oxford, most of the cohort consisted of “health-conscious” persons from England, Wales, Scotland, and Northern Ireland, many of whom were vegetarians. Participants were recruited from the general population in the other centers. The cohorts of France, Norway, Utrecht, and Naples included only women. All study participants provided written informed consent. Ethical approval for this study was obtained from the International Agency for Research on Cancer review board and local participating centers.

Detailed information on the study design, methods, and rationale of the EPIC cohort has been previously reported [13, 14]. Upon recruitment, participants provided medical, dietary, and lifestyle data during interviews and via questionnaires, including information on alcohol use, smoking status, physical activity, education, reproductive history, breastfeeding, hormone use, and previous illnesses. Diet over the previous 12 months was assessed using validated country-specific questionnaires [13], and individual nutrient intakes were derived from foods included in the dietary questionnaires through the standardized EPIC Nutrient Database [15]. To correct for systematic under- or overestimation of dietary intake between the study centers, dietary values across centers were scaled using an additive calibration model [16]. All dietary variables used in this study were calculated by using additive calibration.

For the present study, women with prevalent cancers (except non-melanoma skin cancer) at study enrollment (*n* = 20,477), women who were lost to follow-up (*n* = 2557), pregnant women at study enrollment (*n* = 556), and women with missing data on diet, weight or height, physical activity, or breastfeeding were excluded from the analyses (*n* = 78,245). The latter exclusions due to missing covariates comprised all participants from Umeå, Sweden, and Norway, where physical activity was not collected in sufficient detail as well as participants from Bilthoven, the Netherlands, where information on breastfeeding was not collected. Additionally, in order to exclude implausible dietary intakes, participants within the lowest and highest 1% of the cohort distribution of the ratio of total energy intake to estimated energy requirements were excluded (*n* = 5033). Therefore, 260,151 women were finally included in the analytical sample.

### WCRF/AICR lifestyle score

A score reflecting the adherence to the World Cancer Research Fund/American Institute for Cancer Research (WCRF/AICR) cancer prevention recommendations was constructed, as in previous studies [5, 17, 18]. Of the ten recommendations (components) in the updated WCRF/AICR report [19], the ones pertinent to healthy body weight, physical activity, consumption of plant-based foods, energy-dense foods, red and processed meat, and sugary drinks and alcohol, as well as the recommendation for breastfeeding, were used for the construction of the score (see Additional file 1: Table S1). As in a previous EPIC publication, the recommendation regarding supplement use (due to lack of data) and the separate recommendation pertinent to cancer survivors (not applicable in our study population) were not used for the lifestyle score in the present project [17].

Detailed information on the construction of the WCRF/AICR lifestyle score for the EPIC cohort has previously been reported [17]. In short, a score of 1 was assigned to the corresponding component when a recommendation was met. Intermediate scores (0.5 points) were assigned when recommendations were partially met. The scoring system was constructed such as that each recommendation would contribute equally to the total WCRF/AICR lifestyle score. Since the “eat whole grains, vegetables, fruit, and beans” recommendation contained two sub-recommendations (fruit and vegetable consumption and dietary fiber intake), the component score was calculated as the average of the sub-recommendations’ score. The scores obtained for each component were summed to calculate the WCRF/AICR lifestyle score for each study participant. The score ranged from 0 to 8, and higher scores indicated greater agreement with the WCRF/AICR cancer prevention recommendations. The score was additionally categorized into three groups: category 1 (0 to 3 points), category 2 (> 3 to ≤ 5 points), and category 3 (> 5 points).

The main difference between our WCRF/AICR lifestyle score and the scores used previously in EPIC and other studies [5, 17, 18] is the inclusion of the consumption of sugary drinks as a separate recommendation. Previous studies considered the consumption of sugary drinks as a sub-recommendation, together with the consumption of energy-dense foods (in order to capture the WCRF/AICR recommendation: “Food and drinks that promote weight gain. Limit consumption of energy-dense foods; avoid sugary drinks”). We decided to include the consumption of sugary drinks as a separate recommendation, in order to be in concordance with the updated WCRF/AICR cancer prevention recommendations from 2018 [19] (see Additional file 1: Table S1).

Of note, we decided to assign 1 point for adherence to the WCRF/AICR recommendation on alcohol consumption to individuals, who had reported moderate alcohol consumption (≤ 10 g of ethanol intake per day), in addition to non-consumers [5, 17]. This decision was made to reduce the risk of reverse causality, given that many previous studies have shown “sick quitter effects,” i.e., a higher proportion of persons with impaired health in the non-drinker category [20].

### Outcome assessment

Women who developed first primary incident and histologically confirmed BCIS between recruitment and the latest date of complete information were considered as cases. BCIS cases were identified through population cancer registries in Denmark, Italy, the Netherlands, Spain, Sweden, and the UK. In France, Germany, and Greece, a combination of methods was used including health insurance records, cancer pathology registries, and active follow-up of study participants and their next of kin. The latest dates of complete information for BCIS incidence varied among centers, ranging between 2008 and 2013. Cancer cases were coded according to the 10th Revision of the International Statistical Classification of Diseases, Injuries, and Causes of Death and the Second Revision of the International Classification of Diseases for Oncology.

In situ tumors were further classified based on their morphology as ductal carcinoma in situ (DCIS, morphology code 8500/2) or lobular carcinoma in situ (LCIS, morphology code 8520/2).

### Statistical analyses

Categorical variables were presented by percentages and continuous variables by arithmetic means and standard deviations (SD) for descriptive purposes. Missing covariate values were sporadic (0.1–6.8%, depending on the covariate) and occurred in all participating study centers. We used multiple imputation with a fully conditional specification as provided by PROC MI in SAS [21], and missing values of the covariates (highest level of attained education, smoking status, presence of chronic diseases at recruitment, age at menarche, age at first full-term pregnancy, ever use of oral contraceptive pills, and ever use of menopausal hormone therapy) were imputed on the basis of the distribution of age at recruitment, total dietary energy consumption, menopausal status, and all the individual components of the WCRF/AICR lifestyle score and BCIS diagnosis. Additionally, age at menarche, age at first full-term pregnancy, and ever use of oral contraceptive pills, or ever use of menopausal hormone therapy, were used to impute ever use of menopausal hormone therapy and ever use of oral contraceptive pills, respectively. The PROC MI procedure resulted in five complete data sets. The five complete data sets were then analyzed with Cox proportional hazards regression, and the results for all were combined with PROC MIANALYZE in SAS.

Cox proportional hazards regression was used to assess the association between the WCRF/AICR lifestyle score as well as its individual components and the risk of BCIS development. Entry time was defined as the date of a participant’s recruitment in the study and exit time as the date of diagnosis of BCIS. Participants who were not diagnosed with BCIS were censored on the date of diagnosis of other cancers, except non-melanoma skin cancer, or on the date of loss to follow-up, end of follow-up, or death, whichever came first. All analyses were stratified by age at recruitment (1-year intervals) and center.

Two sets of covariates were evaluated for statistical adjustment. In model 1, we adjusted for the highest level of attained education (none/primary school, technical/secondary school, university degree), smoking status (never smoker, former smoker, current smoker), and total dietary energy consumption (kcal/day). Model 2 was additionally adjusted for a priori determined confounders including the presence of chronic diseases at recruitment (type 2 diabetes, heart disease, and stroke; yes, no), age at menarche (< 12 years, ≥ 12 to ≤ 15 years, > 15 years), age at first full-term pregnancy (nulliparous, < 21 years, ≥ 21 to ≤ 30 years, > 30 years), menopausal status [premenopausal, perimenopausal, postmenopausal (also including surgical postmenopausal)], ever use of oral contraceptive pills (yes, no), and ever use of menopausal hormone therapy (yes, no). The WCRF/AICR lifestyle score was assessed both as a continuous and as a categorical variable. BCIS was evaluated as a composite outcome (i.e., DCIS, LCIS, and all other in situ lesions) and by morphological subtypes (DCIS vs. LCIS). The results are presented as hazard ratios (HR) and corresponding 95% confidence intervals (CI).

Given that BCIS is mainly diagnosed by mammographic screening, we conducted subgroup analyses evaluating the association in centers where most women were recruited via screening programs and in which screening participation during follow-up was high (Utrecht [22] and Florence [23]; screening-recruited cohorts) compared to the other centers. Due to potential differential participation in mammographic screening programs by education level, smoking status, age at recruitment (< 50 vs. ≥ 50 years), and menopausal status, we conducted analyses additionally stratified by these factors. Moreover, given the differential associations observed for some risk factors, especially obesity, by menopausal status and use of menopausal hormone therapy [6], we looked at the association of the individual components of the WCRF/AICR lifestyle score and BCIS risk, stratifying by menopausal status and menopausal hormone therapy use. Sensitivity analyses were conducted, excluding participants with less than 1 year of follow-up (*n* = 1794). Analyses were conducted using SAS version 9.4 (SAS Institute, Cary, NC). All statistical tests were two-sided, and *p* values < 0.05 were considered statistically significant.

## Results

After an overall median follow-up time of 14.9 years, 1277 BCIS (938 DCIS, 96 LCIS, 243 other in situ lesions) cases were diagnosed in the EPIC cohort. Proportions of BCIS to overall breast cancer cases varied between 4.3 and 15.8% across the study centers (Additional file 1: Table S2). As expected, the age-standardized incidence rate of BCIS was higher in the screening-recruited cohorts (EPIC-Florence and EPIC-Utrecht, 15.5 cases per 100,000 person-years) compared to the other centers (10.2 cases per 100,000 person-years), with an overall average incidence rate of 10.5 per 100,000 person-years. A further description of the study population is shown in Table 1. Women from the screening-recruited cohorts were older, less likely to have a university degree, and more likely to be former or current smokers, compared to women recruited in the non-screening centers.

**Table 1:** Baseline characteristics in the total study population

	Total study population (*n* = 260,151)	Screening-recruited cohorts (*n* = 24,727)	Other centers (*n* = 235,424)
WCRF/AICR lifestyle score, mean (SD)	4.5 (1.1)	4.5 (1.1)	4.5 (1.1)
Age at recruitment, mean (SD)	51.7 (9.9)	55.5 (7.2)	51.3 (10.0)
Highest level of attained education, %			
None/primary school	30.4	31.1	30.3
Technical/secondary school	41.7	52.1	40.6
University degree	23.7	16.6	24.4
Unknown/missing	4.2	0.2	4.7
Smoking status, %			
Never smokers	59.1	45.1	60.6
Former smokers	21.8	30.6	20.9
Current smokers	17.5	24.3	16.8
Unknown/missing	1.5	0.0	1.7
Chronic diseases at recruitment, %			
Yes	3.3	4.5	3.2
Unknown	6.8	0.4	7.5
Menopausal status, %			
Premenopausal	33.4	17.6	35.0
Postmenopausal	49.9	65.0	48.3
Perimenopausal	16.8	17.5	16.7
Ever use of oral contraceptive pills, %			
Yes	56.7	57.7	56.6
Unknown	0.4	0.1	0.4
Ever use of menopausal hormone therapy, %			
Yes	24.7	23.6	24.8
Unknown	6.0	0.1	6.6
Age at menarche, %			
< 12 years	15.7	15.6	15.7
≥ 12 to ≤ 15 years	77.1	75.9	77.2
> 15 years	6.2	7.2	6.1
Unknown	1.0	1.3	1.0
Age at first full-term pregnancy, %			
Nulliparous	15.6	13.9	15.8
< 21 years	11.7	6.3	12.3
≥ 21 to ≤ 30 years	63.9	69.1	63.4
> 30 years	8.6	10.7	8.4
Unknown	0.2	0.0	0.2

*SD* standard deviation, *WCRF/AICR* World Cancer Research Fund/American Institute for Cancer Research

The associations between the WCRF/AICR lifestyle score and BCIS risk are shown in Table 2. The WCRF/AICR lifestyle score was not significantly associated with BCIS risk (HR = 0.98, 95% CI 0.93–1.03; per one unit of increase in the WCRF/AICR lifestyle score, HR_cat 3 vs. cat 1_ = 0.98, 95% CI 0.80–1.22; in the multivariable models) in the full EPIC cohort*.* A significant inverse BCIS risk association for WCRF/AICR lifestyle score was observed in the screening-recruited cohorts (HR = 0.85, 95% CI 0.73–0.99; per one unit of increase in the WCRF/AICR lifestyle score in the multivariable model), but not in the other centers (HR = 0.99, 95% CI 0.94–1.05; per one unit of increase in the WCRF/AICR lifestyle score in the multivariable model). While prespecified subgroup analyses of smoking status and menopausal status showed similar associations as the overall analyses, the inverse association between lifestyle score and BCIS risk was more pronounced among women with lower education level in the screening-recruited cohorts (Table 2). Associations between lifestyle score and BCIS risk from analyses stratified by age at recruitment (< 50 vs. ≥ 50 years) were similar in the two subgroups (data not shown). Analyses by morphological subtype showed similar, non-significant associations for DCIS (HR = 0.97, 95% CI 0.91–1.03) or LCIS risk (HR = 0.91, 95% CI 0.75–1.10; per one unit of increase in the WCRF/AICR lifestyle score in the multivariable model for the total study population; Additional file 1: Table S3).

**Table 2:** Associations between the WCRF/AICR lifestyle score and in situ breast cancer risk

	Total study population (*n* = 260,151)			Screening-recruited cohorts (*n* = 24,727)			Other centers (*n* = 235,424)		
	*N* cases/non-cases	Model 1	Model 2	*N* cases/non-cases	Model 1	Model 2	*N* cases/non-cases	Model 1	Model 2
		HR (95% CI)	HR (95% CI)		HR (95% CI)	HR (95% CI)		HR (95% CI)	HR (95% CI)
WCRF/AICR lifestyle score, continuous (0–8)	1277/258,874	0.97 (0.92–1.02)	0.98 (0.93–1.03)	159/24,568	0.83 (0.72–0.97)	0.85 (0.73–0.99)	1118/234,306	0.99 (0.93–1.05)	0.99 (0.94–1.05)
WCRF/AICR lifestyle score 0–3	125/26,684	Ref.	Ref.	20/2426	Ref.	Ref.	105/24,258	Ref.	Ref.
WCRF/AICR lifestyle score > 3–5	811/157,392	1.11 (0.92–1.34)	1.12 (0.93–1.36)	104/14,910	0.81 (0.50–1.31)	0.84 (0.52–1.36)	707/142,482	1.16 (0.95–1.43)	1.17 (0.95–1.45)
WCRF/AICR lifestyle score > 5	341/74,798	0.96 (0.78–1.18)	0.98 (0.80–1.22)	35/7232	0.56 (0.32–0.97)	0.59 (0.33–1.03)	306/67,566	1.04 (0.83–1.30)	1.06 (0.84–1.33)
Education level									
None/primary school	262/79,542	0.91 (0.80–1.02)	0.91 (0.80–1.03)	49/7664	0.74 (0.56–0.97)	0.76 (0.58–1.00)	213/71,878	0.95 (0.83–1.09)	0.95 (0.83–1.09)
Technical/secondary school	596/113,801	0.97 (0.90–1.05)	0.99 (0.91–1.07)	79/12,825	0.86 (0.69–1.06)	0.87 (0.70–1.08)	517/100,976	0.98 (0.91–1.07)	1.00 (0.92–1.09)
University degree	419/65,531	1.00 (0.91–1.09)	1.00 (0.91–1.10)	31/4079	0.93 (0.66–1.31)	0.95 (0.67–1.35)	388/61,452	1.00 (0.91–1.10)	1.01 (0.91–1.11)
Smoking status									
Never smokers	799/155,775	0.99 (0.93–1.06)	1.00 (0.93–1.07)	75/11,065	0.85 (0.68–1.07)	0.85 (0.68–1.06)	724/144,710	1.01 (0.94–1.08)	1.01 (0.94–1.09)
Ever smokers	478/103,099	0.95 (0.87–1.03)	0.96 (0.88–1.05)	84/13,503	0.81 (0.66–0.99)	0.84 (0.68–1.03)	394/89,596	0.98 (0.89–1.07)	0.99 (0.90–1.09)
Menopausal status at recruitment									
Premenopausal	374/86,383	1.05 (0.95–1.15)	1.04 (0.95–1.15)	28/4321	0.90 (0.63–1.28)	0.88 (0.61–1.26)	346/82,062	1.06 (0.96–1.17)	1.06 (0.95–1.17)
Postmenopausal	599/129,150	0.94 (0.87–1.01)	0.94 (0.87–1.02)	95/15,964	0.86 (0.70–1.04)	0.86 (0.71–1.05)	504/113,186	0.95 (0.87–1.03)	0.96 (0.88–1.04)
Perimenopausal	304/43,341	0.95 (0.86–1.06)	0.97 (0.87–1.08)	36/4283	0.78 (0.57–1.06)	0.83 (0.60–1.14)	268/39,058	0.98 (0.87–1.10)	1.00 (0.89–1.12)

Model 1: adjusted for the highest level of attained education (none/primary school, technical/secondary school, university), smoking status (never smoker, former smoker, current smoker), and total energy intake (kcal/day). Model 2: model 1 and additionally adjusted for the presence of chronic diseases at recruitment (yes/no), age at menarche (< 12 years, ≥ 12 to ≤ 15 years, > 15 years), age at first full-term pregnancy (nulliparous, < 21 years, ≥ 21 to ≤ 30 years, > 30 years), menopausal status (premenopausal, perimenopausal, postmenopausal (also including surgical postmenopausal)), ever use of oral contraceptive pills (yes/no), and ever use of menopausal hormone therapy (yes/no). All analyses were stratified for center and age at recruitment (1-year intervals). In subgroup analyses, the corresponding grouping variable was not included as a covariate in the model

*CI* confidence interval, *HR* hazard ratio, *WCRF/AICR* World Cancer Research Fund/American Institute for Cancer Research

The mutually adjusted HRs for BCIS incidence associated with the individual components of the WCRF/AICR lifestyle score are shown in Table 3. In the overall study population, as well as in the analyses stratified by recruitment mode, none of the components of the score was significantly associated with BCIS. In the analyses stratified by menopausal status and menopausal hormone therapy use, BMI was inversely associated with BCIS risk in premenopausal women (Additional file 1: Table S4).

**Table 3:** Associations between adherence to individual WCRF/AICR cancer prevention recommendations and in situ breast cancer risk

WCRF/AICR cancer prevention recommendation*	Total study population (*n* = 260,151)		Screening-recruited cohorts (*n* = 24,727)		Other centers (*n* = 235,424)	
	*N* cases/non-cases	HR (95% CI)	*N* cases/non-cases	HR (95% CI)	*N* cases/non-cases	HR (95% CI)
Limit sugary drinks						
0	189/39,589	Ref.	25/3537	Ref.	164/36,052	Ref.
0.5	799/160,657	0.95 (0.81–1.13)	103/15,276	1.01 (0.64–1.58)	696/145,381	0.94 (0.79–1.13)
1	289/58,628	0.96 (0.78–1.18)	31/5755	0.87 (0.48–1.57)	258/52,873	0.97 (0.77–1.21)
Breastfeed your baby						
0	407/77,037	Ref.	48/6937	Ref.	359/70,100	Ref.
0.5	466/90,454	0.97 (0.82–1.14)	65/9253	1.02 (0.63–1.66)	401/81,201	0.96 (0.80–1.14)
1	404/91,383	0.99 (0.83–1.19)	46/8378	0.80 (0.48–1.35)	358/83,005	1.02 (0.85–1.23)
Limit alcohol						
0	213/34,536	Ref.	37/4610	Ref.	176/29,926	Ref.
0.5	232/44,967	0.88 (0.73–1.06)	28/3820	0.94 (0.57–1.56)	204/41,147	0.88 (0.71–1.08)
1	832/179,371	0.88 (0.75–1.03)	94/16,138	0.75 (0.50–1.12)	738/163,233	0.90 (0.76–1.08)
Limit red and processed meat						
0	530/98,891	Ref.	76/11,309	Ref.	454/87,582	Ref.
0.5	588/115,191	1.04 (0.92–1.18)	68/10,938	1.13 (0.79–1.60)	520/104,253	1.03 (0.90–1.18)
1	159/44,792	0.94 (0.75–1.17)	15/2321	1.31 (0.73–2.35)	144/42,471	0.90 (0.71–1.14)
Eat whole grains, vegetables, fruit, and beans						
0	109/22,992	Ref.	6/735	Ref.	103/22,257	Ref.
0.25	191/36,549	1.07 (0.84–1.37)	18/2763	0.68 (0.26–1.74)	173/33,786	1.11 (0.87–1.43)
0.5	352/76,202	0.88 (0.69–1.12)	58/8787	0.61 (0.25–1.51)	294/67,415	0.90 (0.70–1.16)
0.75	405/79,341	1.00 (0.77–1.29)	50/7893	0.54 (0.21–1.41)	355/71,448	1.06 (0.81–1.39)
1	220/43,790	0.95 (0.70–1.30)	27/4390	0.41 (0.14–1.23)	193/39,400	1.04 (0.75–1.45)
Limit “fast foods”						
0	138/31,684	Ref.	23/3294	Ref.	115/28,390	Ref.
0.5	787/153,617	1.12 (0.91–1.36)	109/16,473	1.21 (0.72–2.02)	678/137,144	1.11 (0.89–1.38)
1	352/73,573	1.01 (0.78–1.32)	27/4801	1.21 (0.59–2.48)	325/68,772	0.99 (0.74–1.30)
Be physically active						
0	589/128,040	Ref.	52/8307	Ref.	537/119,733	Ref.
0.5	257/43,378	1.11 (0.95–1.29)	38/4523	1.30 (0.83–2.02)	219/38,855	1.08 (0.92–1.27)
1	431/87,456	1.00 (0.88–1.15)	69/11,738	0.88 (0.59–1.31)	362/75,718	1.03 (0.90–1.19)
Be a healthy weight						
0	143/40,712	Ref.	22/3310	Ref.	121/37,402	Ref.
0.5	359/76,314	1.15 (0.95–1.40)	61/8668	1.06 (0.65–1.74)	298/67,646	1.16 (0.94–1.44)
1	775/141,848	1.09 (0.90–1.31)	76/12,590	0.87 (0.53–1.43)	699/129,258	1.13 (0.92–1.38)

Model adjusted for the highest level of attained education (none/primary school, technical/secondary school, university), smoking status (never smoker, former smoker, current smoker), total energy intake (kcal/day), presence of chronic diseases at recruitment (yes/no), age at menarche (< 12 years, ≥ 12 to ≤ 15 years, > 15 years), age at first full-term pregnancy (nulliparous, < 21 years, ≥ 21 to ≤ 30 years, > 30 years), menopausal status (premenopausal, perimenopausal, postmenopausal (also including surgical postmenopausal)), ever use of oral contraceptive pills (yes/no), and ever use of menopausal hormone therapy (yes/no). Additionally, all individual components were adjusted for the remaining components of the WCRF/AICR lifestyle score. All analyses were stratified for center and age at recruitment (1-year intervals). *Detailed information on the operationalization of the WCRF/AICR lifestyle score can be found in Additional file 1: Table S1

*CI* confidence interval, *HR* hazard ratio, *WCRF/AICR* World Cancer Research Fund/American Institute for Cancer Research

Sensitivity analyses excluding women with less than 1 year of follow-up, or using the slightly different lifestyle score previously used by Romaguera et al. in the EPIC cohort [17], did not substantially modify our results (data not shown).

## Discussion

In the present large prospective study, we investigated the association between adherence to the WCRF/AICR cancer prevention recommendations and risk of BCIS. Overall, we did not find an association between the WCRF/AICR lifestyle score and BCIS risk. However, subgroup analyses among women who were mostly recruited via mammographic screening programs in the EPIC centers in Florence and Utrecht, with high screening participation rates during follow-up, did show an inverse association.

To the best of our knowledge, even though no other studies exist on lifestyle scores in relation to BCIS risk, our findings are comparable to those of previous studies assessing invasive or total (invasive and in situ tumors as composite endpoint) breast cancer risk. The majority of these studies have reported inverse associations between the WCRF/AICR lifestyle score and invasive [both in postmenopausal and in mixed (i.e., pre- and postmenopausal) populations] [4, 17, 18, 24, 25] or total breast cancer risk [5], with few exceptions [3, 26]. Particularly, those studies on primarily postmenopausal breast cancer risk that recruited participants via mammographic screening programs [18] or adjusted for mammographic screening attendance in their analyses [4] reported associations of comparable magnitude to those found in our study for the screening-recruited EPIC cohorts. Given that BCIS is a mainly screen-detected cancer, the inverse association observed in the screening-recruited cohorts might suggest masking of a similar association in the total population due to screening bias (i.e., women with a more health-conscious lifestyle being more prone to screening participation and screening-detected BCIS) and undetected BCIS status at recruitment. To this end, our overall analyses were limited by the fact that screening participation was not uniformly assessed in most EPIC cohorts.

The lack of a statistically significant association between the individual components of the WCRF/AICR lifestyle score and BCIS risk that we observed in the present study is overall consistent with the findings from studies on invasive breast cancer risk using similar scores [18, 25–27], even though for some individual components (e.g., alcohol consumption) associations have been detected [4, 24, 27]. The inverse association between BMI and BCIS risk in premenopausal women in the present study is consistent with recently published data from a pooling project, in which we took part with EPIC [7]. By contrast, unlike in the NIH-AARP Diet and Health Study [6], we did not observe a significant positive association between BMI and postmenopausal BCIS among never users of menopausal hormone therapy, but our results are in accordance with those of the Million Women Study [8].

Our findings from the subgroup analyses in the screening-recruited EPIC cohorts could suggest that a combined healthy lifestyle (rather than individual components alone) is important for the carcinogenetic process in the breast. However, the lack of association for individual components of the WCRF/AICR lifestyle score in our analyses could also be due to the relatively small proportion of the study population in the screening-recruited EPIC cohorts (9.5% of the total study population). Future large-scale studies with more detailed information on mammographic screening attendance during follow-up and BCIS status at recruitment are needed in order to investigate the individual lifestyle factors in relation to BCIS risk in more detail.

Given the agreement between our subgroup finding of an inverse association between lifestyle score and BCIS risk and those reported in other studies for invasive or total breast cancer (i.e., simultaneously including invasive and in situ tumors) [5, 17, 18, 25], as well as other overlapping risk factors between BCIS and invasive tumors [8], BCIS and invasive breast cancer may be diseases with etiological similarities. Of note, adherence to the WCRF/AICR lifestyle score has been previously associated with lower mammographic density [28], a risk factor for both invasive and in situ tumors. Thus, associations between pre-diagnostic lifestyle and risk of BCIS as well as invasive cancer could suggest that lifestyle acts at a relatively early stage of breast carcinogenesis. Potential mechanisms underlying the associations between lifestyle and breast cancer could be effects of lifestyle on basal inflammation, steroid hormone metabolism, and insulin sensitivity, and studies suggest that people following the WCRF/AICR cancer prevention recommendations have a more favorable biomarker profile (i.e., lower levels of circulating estrogen metabolites, inflammatory cytokines, and C-peptide), compared to those who do not [29, 30].

The strengths of this study included the prospective design and the substantial number of histologically confirmed incident BCIS cases, but it also had some limitations. Information on lifestyle habits was only available from baseline and thus may not reflect the long-term lifestyle habits of the study participants. Furthermore, information on mammographic screening attendance was not available for large parts of our study population. The fact that proportions of BCIS to total breast cancer cases ranged between 4.3 and 15.8% across the centers of the EPIC cohort suggests that screening participation but potentially also BCIS registration were different across study sites. In this context, it should be noted that we could not disentangle the underlying reasons with our data. Temporal and regional differences in BCIS reporting are not expected to be differential among EPIC participants with different lifestyle behaviors or characteristics (e.g., education, smoking status), differential detection bias, i.e., a differential individual likelihood to participate in screening related to individual health consciousness and socio-economic status cannot be ruled out. Nevertheless, we were able to perform analyses restricted to women mainly recruited via organized mammographic screening programs at the EPIC centers in Florence and Utrecht and reported the results from these analyses separately. While our main objective was to evaluate the adherence to lifestyle recommendations in relation to BCIS risk, we acknowledge that more etiology-oriented analyses on BCIS risk from EPIC and other cohorts are needed, also taking into account morphological tumor subtypes based on larger case numbers. Finally, as in any observational study, we cannot exclude the possibility of residual confounding.

## Conclusions

In summary, women with a higher lifestyle score including healthy body weight, regular physical activity, consumption of a high-quality diet, breastfeeding, and drinking a moderate amount of alcohol, if any, did not have a statistically significant lower risk of BCIS compared to participants who did not adhere to these habits in the EPIC cohort. However, there was a statistically significant inverse association among participants recruited via screening programs and with high screening participation during follow-up, suggesting that a true inverse association between lifestyle habits and BCIS risk in the overall cohort may have been masked by the lack of information on screening attendance. The potential inverse association between lifestyle and BCIS risk in our subgroup analyses restricted to screening-recruited EPIC cohorts is consistent with the inverse associations between lifestyle scores and mammographic density as well as breast cancer risk reported in previous studies. These findings may indicate that BCIS and invasive breast cancer have a similar risk factor profile. Additional studies, with more detailed information on screening participation, are required in order to validate our findings.

## Supplementary information

**Additional file 1: **This file contains **Table S1.** The World Cancer Research Fund/American Institute for Cancer Research (WCRF/AICR) cancer prevention recommendations and the operationalization of the WCRF/AICR lifestyle score in the European Prospective Investigation into Cancer and Nutrition cohort; **Table S2.** The proportion of primary in situ breast cancer cases over the total primary breast cancer cases diagnosed in the European Prospective Investigation into Cancer and Nutrition cohort; **Table S3.** The association between the World Cancer Research Fund/American Institute for Cancer Research lifestyle score and the risk for the most common morphological subtypes of BCIS (in the total study population and according to recruitment mode) in the European Prospective Investigation into Cancer and Nutrition cohort; **Table S4.** The associations between the individual components of the World Cancer Research Fund/American Institute for Cancer Research lifestyle score and in situ breast cancer risk by menopausal status and menopausal hormone use assessed in the total study population.

## Data Availability

For information on how to submit an application for gaining access to EPIC data and/or biospecimens, please follow the instructions at http://epic.iarc.fr/access/index.php.

## Abbreviations

AICR: American Institute for Cancer Research
BCIS: In situ breast cancer
BMI: Body mass index
CI: Confidence interval
EPIC: European Prospective Investigation into Cancer and Nutrition
HR: Hazard ratio
WCRF: World Cancer Research Fund
