# Supplementary material for: Adherence to the World Cancer Research Fund/American Institute for Cancer Research cancer prevention recommendations and risk of in situ breast cancer in the European Prospective Investigation into Cancer and Nutrition (EPIC) cohort
Source: BMC Med. 2019 Dec 2;17:221. doi: 10.1186/s12916-019-1444-0 (PMC6886197; doi:10.1186/s12916-019-1444-0)
Supplement: Supplementary file 1 — Additional file 1: This file contains Table S1. The World Cancer Research Fund/American Institute for Cancer Research (WCRF/AICR) cancer prevention recommendations and the operationalization of the WCRF/AICR lifestyle score in the European Prospective Investigation into Cancer and Nutrition cohort; Table S2. The proportion of primary in situ breast cancer cases over the total primary breast cancer cases diagnosed in the European Prospective Investigation into Cancer and Nutrition cohort; Table S3. The association between the World Cancer Research Fund/American Institute for Cancer Research lifestyle score and the risk for the most common morphological subtypes of BCIS (in the total study population and according to recruitment mode) in the European Prospective Investigation into Cancer and Nutrition cohort; Table S4. The associations between the individual components of the World Cancer Research Fund/American Institute for Cancer Research lifestyle score and in situ breast cancer risk by menopausal status and menopausal hormone use assessed in the total study population. [file 12916_2019_1444_MOESM1_ESM.pdf]

Table S1. World Cancer Research Fund/ American Institute for Cancer Research (WCRF/AICR) cancer prevention recommendations and operationalization of the WCRF/AICR lifestyle score in the European Prospective Investigation into Cancer and Nutrition (EPIC) cohort [1]

WCRF/AICR cancer prevention recommendation	Operationalization	Scoring
Be a healthy weight	- BMI: 18.5 - 24.9 kg/m^2	1
	- BMI: 25.0 - 29.9 kg/m^2	0.5
	- BMI <18.5 or BMI >30.0 kg/m^2	0
Be physically active	- Manual/heavy manual job, or >2 h/wk of vigorous PA or >30 min/d of cycling/sports	1
	- 15-30 min/d of cycling/sports	0.5
	- <15 min/d of cycling/sports	0
Eat wholegrains, vegetables, fruit, beans	- Fruits and vegetables ≥400 g/d	1
	- 200≤ Fruits and vegetables <400 g/d	0.5
	- Fruits and vegetables <200 g/d	0
	- Dietary fiber ≥25 g/d	1
	- 12.5≤ Dietary fiber <25 g/d	0.5
	- Dietary fiber <12.5 g/d	0
Limit 'fast foods'	- ED ≤ 125 kcal/100 g/d	1
	- 125≤ ED≤ 175 kcal/100 g/d	0.5
	- ED>175 kcal/100 g/d	0
Limit red & processed meat	- Red and processed meat <500 g/wk and processed meat intake <3 g/d	1
	- Red and processed meat <500 g/wk and	0.5
	- 3 < processed meat intake <50 g/d	
	- Red and processed meat >500 g/wk or processed meat intake ≥50 g/d	0
Limit sugary drinks	- Sugary drinks: 0 g/d	1
	- 0< Sugary drinks ≤250 g/d	0.5
	- Sugary drinks >250 g/d	0
Limit alcohol	- Ethanol intake ≤10 g/d	1
	- 10< Ethanol intake ≤20 g/d	0.5
	- Ethanol intake >20 g/d	0
Don't rely on supplements	Not applicable to this population	Not applicable to this population
Breastfeed your baby	- Cumulative breastfeeding ≥6 months	1
	- 0< Cumulative breastfeeding <6 months	0.5
	- No breastfeeding	0
After a cancer diagnosis	Not applicable to this population	Not applicable to this population

[1] ED: Energy density. ED was calculated as energy (kcal) from foods (solid, semi-solid and liquid foods) divided by the weights (g) of these foods. Drinks were not included in the calculation. Abbreviations: BMI: Body mass index; g/d: grams per day; g/wk: grams per week; min/day: minutes per day; h/wk: hours per week

Table S2. The proportion of primary in situ breast cancer cases over the total primary breast cancer cases diagnosed in the European Prospective Investigation into Cancer and Nutrition (EPIC) cohort

Country/ Center	Primary in situ breast cancer cases	Total primary breast cancer cases[1]	Proportion primary in situ breast cancer / total primary breast cancer cases %
**France**	439	3,365	13.0
Ile-de-France	85	684	12.4
North-West of France	67	592	11.3
North-East of France	93	736	12.6
Rhone-Alpes/Auvergne	66	419	15.8
Provence/Languedoc	62	473	13.1
South-West of France	66	461	14.3
**Italy**	116	1,316	8.8
Florence	57	467	12.2
Varese	26	414	6.3
Ragusa	5	106	4.7
Turin	21	179	11.7
Naples	7	150	4.7
**Spain**	69	704	9.8
Asturias	18	136	13.2
Granada	10	142	7.0
Murcia	6	141	4.3
Navarra	14	147	9.5
San Sebastian	21	138	15.2
**United Kingdom**	256	1,964	13.0
Cambridge	67	504	13.3
Oxford	189	1460	12.9
**The Netherlands**			
Utrecht	102	832	12.3
**Greece**	16	256	6.3
**Germany**	93	899	10.3
Heidelberg	49	505	9.7
Potsdam	44	394	11.2
**Sweden**			
Malmo	66	902	7.3
**Denmark**	120	1,951	6.2
Aarhus	25	512	4.9
Copenhagen	95	1,439	6.6

[1] Total breast cancer cases include all primary in situ breast cancers and all primary invasive breast cancers diagnosed in the EPIC cohort.

Table S3. Association between the World Cancer Research Fund/ American Institute for Cancer Research (WCRF/AICR) lifestyle score and risk for the most common morphological subtypes of in situ breast cancer in the total study population and according to recruitment mode in the European Prospective Investigation into Cancer and Nutrition (EPIC) cohort [1]

	Total study population (n=260,151)			Screening-recruited cohorts (n=24,727)			Other centers (n=235,424)		
		Model 1	Model 2		Model 1	Model 2		Model 1	Model 2
	N cases/ non-cases	HR (95% CI)	HR (95% CI)	N cases/ non-cases	HR (95% CI)	HR (95% CI)	N cases/ non-cases	HR (95% CI)	HR (95% CI)
Ductal Carcinoma In Situ									
WCRF/AICR lifestyle score, continuous (0-8)	938/259,213	0.96 (0.90-1.02)	0.97 (0.91-1.03)	129/24,598	0.86 (0.73-1.01)	0.87 (0.74-1.04)	809/234,615	0.98 (0.92-1.05)	0.99 (0.92-1.05)
WCRF/AICR lifestyle score 0-3	91/26,718	Ref.	Ref.	16/2,430	Ref.	Ref.	75/24,288	Ref.	Ref.
WCRF/AICR lifestyle score >3-5	603/157,600	1.13 (0.90-1.41)	1.14 (0.91-1.43)	85/14,929	0.83 (0.48-1.42)	0.86 (0.50-1.48)	518/142,671	1.19 (0.93-1.52)	1.20 (0.94-1.53)
WCRF/AICR lifestyle score >5	244/74,895	0.94 (0.74-1.21)	0.97 (0.75-1.24)	28/7,239	0.56 (0.30-1.05)	0.60 (0.32-1.13)	216/67,656	1.03 (0.79-1.34)	1.05 (0.80-1.37)
Lobular Carcinoma In Situ									
WCRF/AICR lifestyle score, continuous (0-8)	96/260,055	0.90 (0.74-1.08)	0.91 (0.75-1.10)	4/24,723	0.99 (0.38-2.58)	0.78 (0.27-2.26)	92/235,332	0.89 (0.74-1.08)	0.90 (0.74-1.10)
WCRF/AICR lifestyle score 0-3	14/26,795	Ref.	Ref.		Ref.	Ref.	14/24,349	Ref.	Ref.
WCRF/AICR lifestyle score >3-5	55/158,148	0.72 (0.39-1.32)	0.74 (0.40-1.35)		-	-	52/143,137	0.67 (0.37-1.25)	0.69 (0.37-1.28)
WCRF/AICR lifestyle score >5	27/75,112	0.69 (0.35-1.35)	0.71 (0.36-1.42)		-	-	26/67,846	0.66 (0.33-1.30)	0.68 (0.34-1.36)

[1] Model 1: Adjusted for highest level of attained education (none/primary school, technical /secondary school, university), smoking status (never smoker, former smoker, current smoker) and total energy intake (kcal/d); Model 2: Model 1 and additionally adjusted for presence of chronic diseases at recruitment (yes/no), age at menarche (<12y, ≥12 to ≤15y, >15y), age at first full-term pregnancy (<21y, ≥21 to ≤30y, >30y), menopausal status (premenopausal, perimenopausal, postmenopausal (also including surgical postmenopausal)), ever use of oral contraceptive pills (yes/no) and ever use of menopausal hormone therapy (yes/no). All analyses were stratified for center and age at recruitment (1-year intervals). Due to the low number of lobular carcinoma in situ tumors diagnosed in the screening recruited cohorts, analyses using the WCRF/AICR lifestyle score categories could not be carried out. Abbreviations: CI: confidence interval; HR: hazard ratio

Table S4. Associations between the individual components of the World Cancer Research Fund/ American Institute for Cancer Research (WCRF/AICR) lifestyle score and in situ breast cancer risk by menopausal status and menopausal hormone use in the total study population (n= 260,151) [1]

	Premenopausal (n=86,757)		Post or peri-menopausal never users (n=106,592)		Post or peri-menopausal ever users (n=66,802)	
WCRF/AICR cancer prevention recommendation *	Number of cases/ non-cases	HR (95% CI)	Number of cases/ non-cases	HR (95% CI)	Number of cases/ non-cases	HR (95% CI)
Limit sugary drinks						
0	58/16,546	Ref.	57/12,887	Ref.	74/10,156	Ref.
0.5	235/51,976	1.01 (0.74-1.37)	294/66,707	1.03 (0.76-1.38)	270/41,974	0.82 (0.63-1.08)
1	81/17,861	1.02 (0.68-1.52)	125/26,521	1.11 (0.78-1.59)	83/14,246	0.77 (0.53-1.10)
Breastfeed your baby						
0	121/30,422	Ref.	150/28,231	Ref.	136/18,384	Ref.
0.5	134/29,585	1.00 (0.73-1.38)	171/35,021	0.94 (0.72-1.22)	161/25,848	0.99 (0.75-1.32)
1	119/26,376	0.97 (0.69-1.37)	155/42,863	0.99 (0.74-1.31)	130/22,144	1.02 (0.75-1.39)
Limit alcohol						
0	51/10,286	Ref.	84/13,550	Ref.	78/10,700	Ref.
0.5	63/14,983	0.95 (0.65-1.38)	80/17,014	0.79 (0.58-1.09)	89/12,970	0.93 (0.68-1.26)
1	260/61,114	1.06 (0.77-1.46)	312/75,552	0.79 (0.61-1.03)	260/42,705	0.84 (0.64-1.10)
Limit red & processed meat						
0	149/30,710	Ref.	215/40,071	Ref.	166/28,110	Ref.
0.5	151/34,665	0.95 (0.74-1.22)	206/48,462	0.91 (0.74-1.12)	231/32,064	1.31 (1.05-.163)
1	74/21,008	0.91 (0.62-1.34)	56/17,582	1.01 (0.71-1.43)	29/6,202	0.87 (0.56-1.33)
Eat wholegrains, vegetables, fruit, beans						
0	39/7,758	Ref.	37/9,639	Ref.	33/5,595	Ref.
0.25	58/12,299	0.94 (0.62-1.44)	68/14,840	1.12 (0.74-1.71)	65/9,410	1.22 (0.79-1.90)
0.5	95/25,259	0.70 (0.46-1.07)	132/30,582	0.94 (0.62-1.42)	125/20,361	1.01 (0.66-1.57)
0.75	114/26,547	0.82 (0.52-1.30)	153/33,099	1.04 (0.67-1.62)	138/19,695	1.19 (0.74-1.89)
1	68/14,520	0.84 (0.48-1.48)	85/17,955	1.04 (0.61-1.77)	67/11,315	1.03 (0.58-1.81)
Limit 'fast foods'						

0	57/12,670	Ref.	40/11,285	Ref.	41/7,729	Ref.
0.5	222/50,973	0.91 (0.66-1.28)	298/62,674	1.36 (0.94-1.97)	267/39,970	1.15 (0.79-1.66)
1	95/22,740	0.88 (0.55-1.40)	138/32,156	1.32 (0.84-2.08)	119/18,677	0.94 (0.59-1.49)
Be physically active						
0	167/41,601	Ref.	229/56,207	Ref.	193/30,232	Ref.
0.5	74/15,128	1.14 (0.86-1.51)	85/16,124	1.01 (0.78-1.31)	98/12,126	1.20 (0.93-1.54)
1	133/29,654	1.22 (0.96-1.56)	163/33,785	0.97 (0.78-1.21)	135/24,017	0.90 (0.71-1.13)
Be a healthy weight						
0	28/11,136	Ref.	75/21,344	Ref.	40/8,232	Ref.
0.5	80/20,687	1.50 (0.97-2.32)	147/35,152	0.93 (0.70-1.24)	132/20,475	1.24 (0.86-1.77)
1	266/54,560	1.61 (1.08-2.41)	254/49,620	0.85 (0.65-1.12)	255/37,668	1.07 (0.76-1.52)

[1] Models adjusted for highest level of attained education (none/primary school, technical /secondary school, university), smoking status (never smoker, former smoker, current smoker), total energy intake (kcal/d), the remaining components of the WCRF/AICR score, the presence of chronic diseases at baseline (yes/no), age at menarche (<12y, ≥12 to ≤15y, >15y), age at first full-term pregnancy (<21y, ≥21 to ≤30y, >30y), and ever use of oral contraceptive pills (yes/no). All analyses were stratified for center and age at recruitment (1-year intervals). The case/non-case numbers presented are estimated as the average of the 5 imputed datasets. *Detailed information on the operationalization of the WCRF/AICR lifestyle score can be found in Additional file 1: Table S1. Abbreviations: CI: confidence interval; HR: hazard ratio
